# Isoalantolactone Increases the Sensitivity of Prostate Cancer Cells to Cisplatin Treatment by Inducing Oxidative Stress

**DOI:** 10.3389/fcell.2021.632779

**Published:** 2021-04-20

**Authors:** Hang Huang, Ping Li, Xueting Ye, Fangyi Zhang, Qi Lin, Keming Wu, Wei Chen

**Affiliations:** Department of Urology, The First Affiliated Hospital of Wenzhou Medical University, Wenzhou, China

**Keywords:** prostate cancer, isoalantolactone, cisplatin, oxidative stress, endoplasmic reticulum stress

## Abstract

Prostate cancer is the most common malignancy among men worldwide. Platinum (II)-based chemotherapy has been used to treat a number of malignancies including prostate cancer. However, the potential of cisplatin for treating prostate cancer is restricted owing to its limited efficacy and toxic side effects. Combination therapies have been proposed to increase the efficacy and reduce the toxic side effects. In the present study, we investigated how isoalantolactone (IATL), a sesquiterpene lactone extracted from the medicinal plant *Inula helenium* L., acts synergistically with cisplatin on human prostate cancer cells. We show that IATL significantly increased cisplatin-induced growth suppression and apoptosis in human prostate cancer cells. Mechanistically, the combined treatment resulted in an excessive accumulation of intracellular reactive oxygen species (ROS), which leads to the activation of endoplasmic reticulum (ER) stress and the JNK signaling pathway in human prostate cancer cells. Pretreatment of cells with the ROS scavenger N-acetylcysteine (NAC) significantly abrogated the combined treatment-induced ROS accumulation and cell apoptosis. In addition, the activation of ER stress and the JNK signaling pathway prompted by IATL and cisplatin was also reversed by NAC pretreatment. *In vivo*, we found that IATL combined with cisplatin showed the strongest antitumor effects compared with single agents. These results support the notion that IATL and cisplatin combinational treatment may be more effective for treating prostate cancer than cisplatin alone.

## Introduction

Prostate cancer is the most common malignancy found in men. Current therapy for prostate cancer mainly includes surgery, androgen deprivation therapy, and chemotherapy (Bray et al., 2018; Wilkins et al., 2020). Although androgen deprivation therapy is effective in the initial stages, most patients eventually progress to metastatic castration-resistant prostate cancer, for which there is no effective strategy and is generally calamitous. In addition, there are still many prostate cancer patients who are not sensitive to androgen deprivation therapy (Watson et al., 2015; Powers et al., 2020). Hence, new effective therapeutics for metastatic castration-resistant prostate cancer is urgently needed.

Chemotherapy strategy is another effective and widely used treatment for advanced prostate cancer (Pignot et al., 2018). As one of the most effective chemotherapeutic drugs, cisplatin has been used to treat many types of cancer, including breast cancer, lung cancer, ovarian cancer, and prostate cancer (Hager et al., 2016; Rottenberg et al., 2020). Despite the broad application of cisplatin in cancer treatment, it is associated with serious side effects and drug resistance, which limit its therapeutic efficiency in prostate cancer (Morigi et al., 2015; Su et al., 2019). Combination therapies have been proposed to reduce the adverse effects and drug resistance in recent years (Valentovic, 2018; Ruiz de Porras et al., 2020). Therefore, the identification of new agents to synergize with cisplatin and reduce its adverse effects is needed.

Isoalantolactone (IATL), a sesquiterpene lactone compound isolated from the root of *Inula helenium* L., has been used in traditional Chinese herbal medicine (Kumar et al., 2014). It has been shown that IATL can interrupt the NF-κB/COX-2-mediated signaling cascade and induce apoptosis in glioblastoma (Xing et al., 2019). In addition, various molecules and signal transduction pathways, including p53, death receptor 5, and MAPK, are involved in the process of IATL-induced cell death (Li et al., 2016; Jin et al., 2017; Lu et al., 2018). Importantly, it has been shown that IATL exerts a desirable effect and does not cause serious injury to normal tissues (Xu et al., 2015). Recently, we demonstrated that IATL induced apoptosis via the activation of ROS-mediated ER stress in prostate cancer cells (Chen et al., 2018). However, the synergistic effects of IATL and cisplatin in prostate cancer cells remain unclear. In the present study, we investigated the synergistic effects of IATL and cisplatin in prostate cancer cells and explored the underlying molecular mechanisms involved.

## Materials and Methods

### Cell Culture and Reagents

Human prostate cancer cells DU145 and PC-3 were obtained from the Institute of Biochemistry and Cell Biology, Chinese Academy of Sciences. The cells were cultured in an RPMI 1640 medium containing 10% fetal bovine serum and maintained in an atmosphere of 5% CO_2_ at 37°C. Isoalantolactone (IATL) was purchased from Chengdu Herbpurify Co., Ltd. (Chengdu, China). SP600125 and N-acetylcysteine (NAC) were obtained from Selleck Chemicals (Houston, TX, United States). For Western blot analysis, the antibodies against p-eIF2α, eIF2α, ATF4, CHOP, p-JNK, JNK, and GAPDH were purchased from Cell Signaling Technology (Danvers, MA, United States).

### Cell Viability Assay

Prostate cancer cells (8,000 per well) were seeded on a 96-well plate. Then the cells were treated with the indicated chemical agents for 24 h. DMSO was used as a vehicle. Cell viability was analyzed by a 3-(4,5-dimethylthiazol-2-yl)-2,5-diphenyltetrazolium bromide (MTT) assay. To investigate the synergistic effects between IATL and cisplatin, the combination index (CI) was calculated according to the Chou–Talalay method (Chou, 2010).

### Cell Apoptosis Analysis

Cells were treated with the indicated chemical agents for 24 h. Then the cells were staining with FITC conjugated Annexin V and Propidium Iodide (BD Pharmingen, NJ, United States) in a binding buffer for 30 min according to the manufacturer’s instructions. Cells were collected and analyzed by the FACSCalibur flow cytometer.

### Western Blot Analysis

Cells were collected and lysed in an ice-cold lysis buffer. Protein concentration was determined by using the Bradford protein assay (Bio-Rad, CA, United States). Protein samples were separated by SDS-PAGE and then transferred onto polyvinylidene difluoride (PVDF) membranes. The blotted membrane was blocked with 5% skim milk for 2 h at room temperature and incubated overnight at 4°C with specific primary antibody. Following three washes with TBST, the membrane was incubated with secondary antibody at a 1:2,000 dilution for 1 h. The immunoreactive band was visualized by using an ECL kit.

### Measurement of Cellular ROS Levels

Cellular ROS levels were detected as described previously (Chen et al., 2018). Briefly, cells were treated with the indicated chemical agents for 2 h. Then the cells were stained with 10 μM DCFH-DA (Beyotime, Shanghai, China) for 30 min at 37°C and analyzed by the FACSCalibur flow cytometer.

### Quantitative RT-PCR

Cells were treated with the indicated chemical agents for 6 h. Then the cells were harvested and RNAs were extracted using a Trizol reagent (Invitrogen, CA, United States) according to the manufacturer’s instructions. The expression levels of CHOP were determined as described previously (Chen et al., 2018).

### Measurement of Caspase-3/9 Activity

Cells were treated with the indicated chemical agents for 20 h. The detection of Caspase-3 and Caspase-9 activity was performed using a Caspase-3 and Caspase-9 Activity Assay Kit (Beyotime, Shanghai, China).

### Transfection of siRNA

Knockdown of CHOP was achieved by transfecting siRNA targeting CHOP with the corresponding scrambled siRNA as the negative control (Invitrogen, CA, United States). Transfection was performed using Lipofectamine 3000 (Invitrogen, CA, United States) according to the manufacturer’s instructions as described previously (Chen et al., 2018).

### Xenograft Experiments

Animal experiments were performed according to the Institutional Animal Care and Use Committee (IACUC) guidelines, Wenzhou Medical University. Athymic nude mice (nu/nu, 5-weeks-old, male) were used in this study. Cells (5 × 10^6^) were inoculated subcutaneously into the right flank region of nude mice. The mice were randomized into four groups (*n* = 6) and were treated with vehicle, IATL, cisplatin, or the combination of IATL and cisplatin by intraperitoneal (i.p.) injection once every 3 days at the indicated doses (McPherson et al., 2009; Chen et al., 2018). The tumor volumes were determined by measuring length (l) and width (w) and calculated by the following formula: volume (mm^3^) = length × width^2^/2 at the indicated time points.

### Malondialdehyde (MDA) Assay

For the MDA assay, the tissue samples were prepared according to the manufacturer’s instructions of the Lipid Peroxidation MDA assay kit (Beyotime, Shanghai, China). The MDA levels in tumor tissues were detected by a microplate reader (SpectraMax M5, Molecular Devices, United States).

### Quantification and Statistical Analysis

Data are shown as mean ± standard error of the mean. Statistical significance was determined using Student’s *t*-test. GraphPad Prism 5.0 software was used for statistical analysis, and a *p*-value lower than 0.05 was considered statistically significant.

## Results

### IATL and Cisplatin Synergistically Induce Apoptosis in Prostate Cancer Cells

**FIGURE 1:**
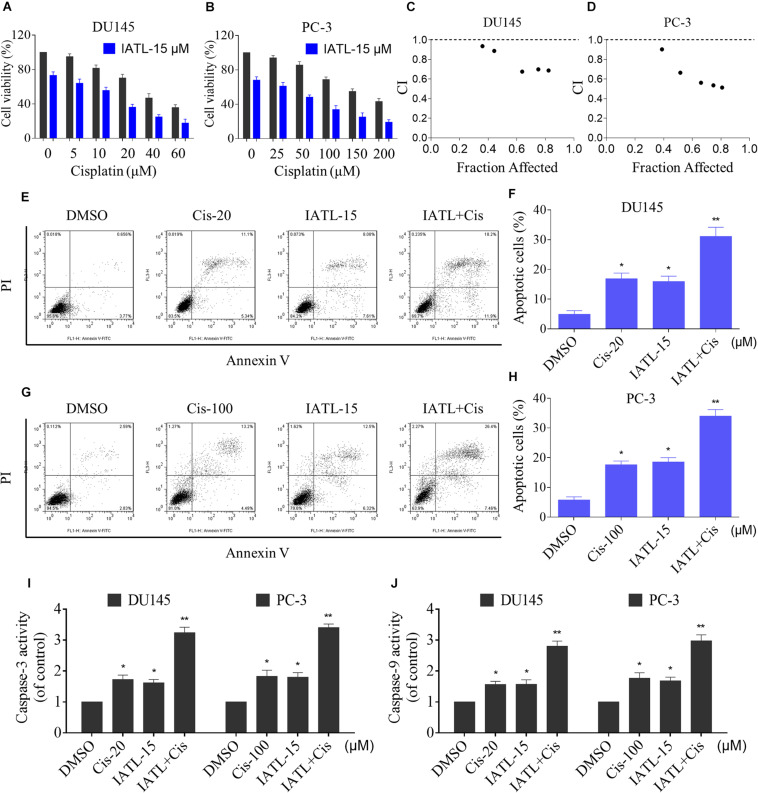
IATL and cisplatin synergistically inhibit cells growth and induce apoptosis. **(A,B)** DU145 or PC-3 cells were treated with IATL or cisplatin alone or their combination at the indicated concentration for 24 h; cell viability was determined by an MTT assay. **(C,D)** Combination index (CI) values were calculated by the Calcusyn software, and a CI value of less than 1 indicates synergism. **(E–H)** DU145 or PC-3 cells were treated with IATL or cisplatin alone or their combination at the indicated concentration for 24 h; cell apoptosis rate was analyzed by flow cytometry. **(I,J)** DU145 or PC-3 cells were treated with IATL or cisplatin alone or their combination at the indicated concentration for 20 h; caspase-3 and caspase-9 activity were measured by an assay kit. *p < 0.05, **p < 0.01.

We first evaluated the effect of the combination of IATL and cisplatin on DU145 and PC-3 cell lines. The MTT assay showed that cisplatin inhibited cell growth as monotherapy, but the inhibitory effect was stronger when in combination with IATL (Figures 1A,B). The combination index (CI) analysis suggested that IATL and cisplatin acted synergistically in DU145 and PC-3 cells (*CI* < 1) (Figures 1C,D). To further investigate the synergistic effect of IATL and cisplatin, the cell apoptosis rate was determined by annexin V/PI staining. The combination of IATL and cisplatin significantly increased apoptotic cell death in both DU145 and PC-3 cells as compared with the agent alone (Figures 1E–H). Furthermore, IATL in combination with cisplatin significantly increased the activity of caspase-3 and caspase-9 (Figures 1I,J). These data indicate that IATL synergized the chemotherapeutic effects of cisplatin in prostate cancer cells.

### IATL and Cisplatin Combination Triggers ROS-Dependent Apoptosis in Prostate Cancer Cells

**FIGURE 2:**
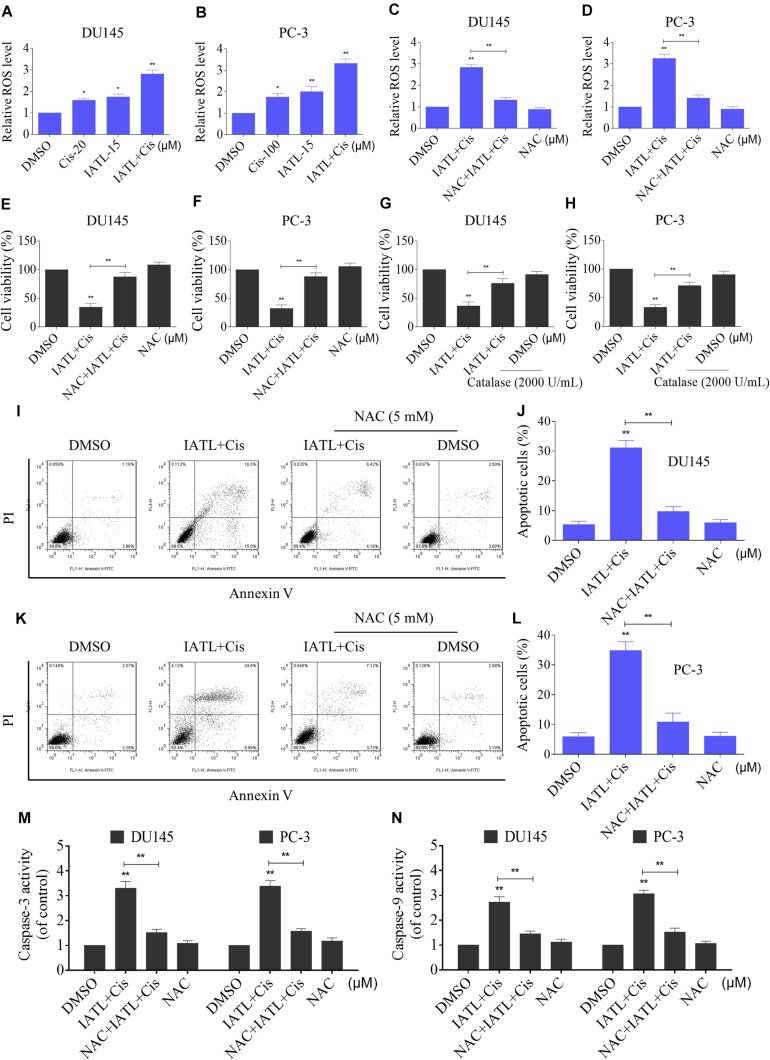
IATL and cisplatin combination triggers ROS-dependent apoptosis in prostate cancer cells. **(A,B)** DU145 or PC-3 cells were treated with IATL or cisplatin alone or their combination at the indicated concentration for 2 h; cellular ROS levels were detected by using the DCFH-DA probe. **(C,D)** DU145 or PC-3 cells were preincubated with NAC (5 mM) for 2 h before exposure to the combination of IATL and cisplatin for 2 h; cellular ROS levels were detected by using the DCFH-DA probe. **(E,F)** DU145 or PC-3 cells were preincubated with NAC (5 mM) for 2 h; cell viability was detected by an MTT assay after being treated with the combination of IATL and cisplatin for 24 h. **(G,H)** DU145 or PC-3 cells were preincubated with catalase (2,000 U/mL) for 2 h; cell viability was detected by an MTT assay after being treated with the combination of IATL and cisplatin for 24 h. **(I–L)** DU145 or PC-3 cells were preincubated with NAC (5 mM) for 2 h; the cell apoptosis rate was detected by flow cytometry after being treated with the combination of IATL and cisplatin for 24 h. **(M,N)** DU145 or PC-3 cells were preincubated with NAC (5 mM) for 2 h; caspase-3 and caspase-9 activity was measured by an assay kit after being treated with the combination of IATL and cisplatin for 20 h. *p < 0.05, **p < 0.01.

Recent studies have shown that ROS accumulation plays a critical role in the apoptosis of cancer cells induced by therapeutic agents (Cho et al., 2020; Hong et al., 2020). Cisplatin is known to induce apoptosis in cancer cells via inducing ROS accumulation (Wang et al., 2020). We therefore assessed the levels of ROS in DU145 and PC-3 cells. The results showed that IATL and cisplatin combination significantly increased ROS levels in both DU145 and PC-3 cells as compared with the agent alone (Figures 2A,B). The ROS scavenger NAC was used to investigate the role of ROS in the synergistic effects of IATL and cisplatin. The results showed that NAC could significantly reverse the combined treatment-induced accumulation of ROS (Figures 2C,D). Importantly, pretreatment with NAC markedly reversed the combined treatment-induced cell growth inhibition in both DU145 and PC-3 cells (Figures 2E,F). Catalase, another ROS scavenger, also abolished the growth inhibitory effect of IATL and cisplatin (Figures 2G,H). Furthermore, pretreatment with NAC markedly reversed the combined treatment-induced apoptosis and activation of caspase-3 and caspase-9 in both DU145 and PC-3 cells (Figures 2I–N). These data suggest that ROS accumulation is crucial for the synergistic effects of IATL and cisplatin.

### IATL and Cisplatin Co-operate to Activate ER Stress

**FIGURE 3:**
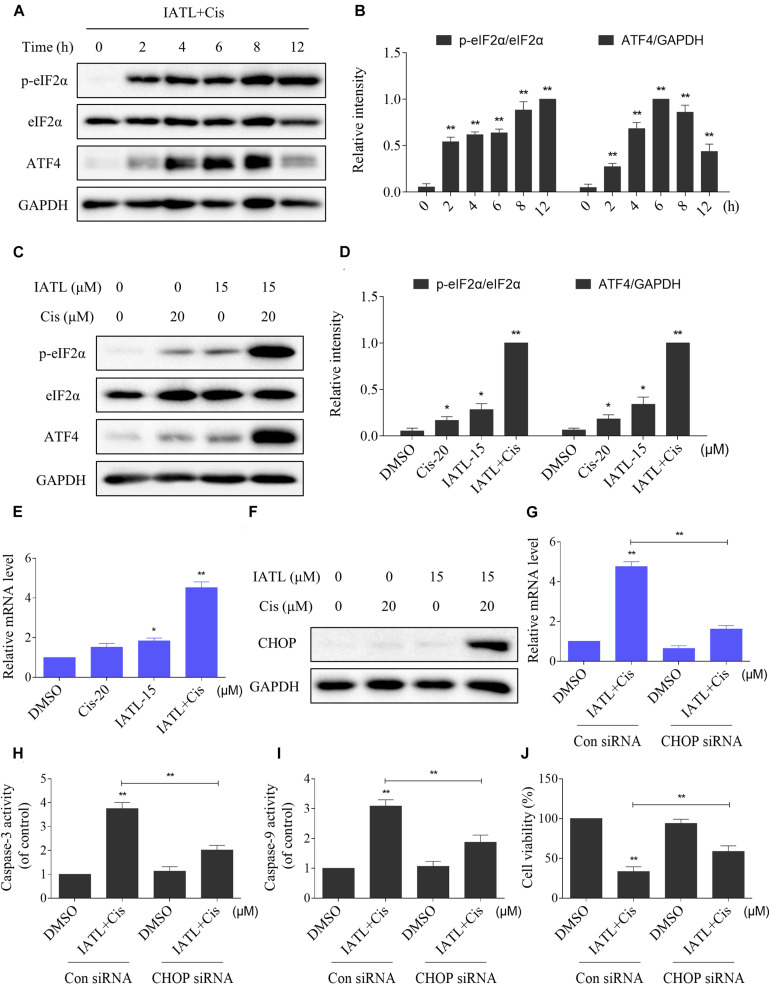
IATL and cisplatin co-operate to activate ER stress in prostate cancer cells. **(A,B)** DU145 cells were treated with IATL (15 μM) and cisplatin (20 μM) combination for the indicated time periods; the expression of p-eIF2α and ATF4 was measured by Western blot. **(C,D)** DU145 cells were treated with IATL (15 μM) or cisplatin (20 μM) alone or their combination (15 μM IATL and 20 μM cisplatin); the expression of p-eIF2α and ATF4 was measured by Western blot. **(E)** DU145 cells were treated with IATL (15 μM) or cisplatin (20 μM) alone or their combination (15 μM IATL and 20 μM cisplatin) for 6 h; the mRNA level of CHOP was determined by qRT-PCR. **(F)** DU145 cells were treated with IATL (15 μM) or cisplatin (20 μM) alone or their combination (15 μM IATL and 20 μM cisplatin) for 12 h; the expression of CHOP was measured by Western blot. **(G)** DU145 cells were infected with CHOP siRNA; the mRNA level of CHOP was determined by qRT-PCR after being treated with the combination of IATL (15 μM) and cisplatin (20 μM) for 6 h. **(H,I)** DU145 cells transfected with CHOP siRNA were treated with the combination of IATL (15 μM) and cisplatin (20 μM) for 20 h; caspase-3 and caspase-9 activity was measured by an assay kit. **(J)** DU145 cells transfected with CHOP siRNA were treated with the combination of IATL (15 μM) and cisplatin (20 μM) for 24 h; cell viability was measured by an MTT assay. *p < 0.05, **p < 0.01.

ER stress is considered as one of the mechanisms contributing to ROS-mediated cell death (Qi et al., 2020; Zhao et al., 2020). Therefore, we measured the levels of ER stress-related proteins in DU145 cells. The time-course experiment showed that the combined treatment significantly increased the expression levels of p-eIF2α and ATF4 (Figures 3A,B). Moreover, the addition of IATL in combination with cisplatin resulted in more significant increase in the expression levels of p-eIF2α and ATF4 as compared with the agent alone (Figures 3C,D). CCAAT/enhancer-binding protein homologous protein (CHOP) is considered as a key mediator of ER stress-induced apoptosis. The results showed that IATL and cisplatin combination significantly increased the mRNA and protein levels of CHOP (Figures 3E,F). Next, we investigated whether CHOP was involved in the antitumor effects of the combination of IATL and cisplatin. The results showed that knockdown of CHOP reversed the combined treatment-induced cell death as well as the activation of caspase-3 and caspase-9 (Figures 3G–J). These results indicate that IATL and cisplatin combination treatment induces cell death via the activation of ER stress.

**FIGURE 4:**
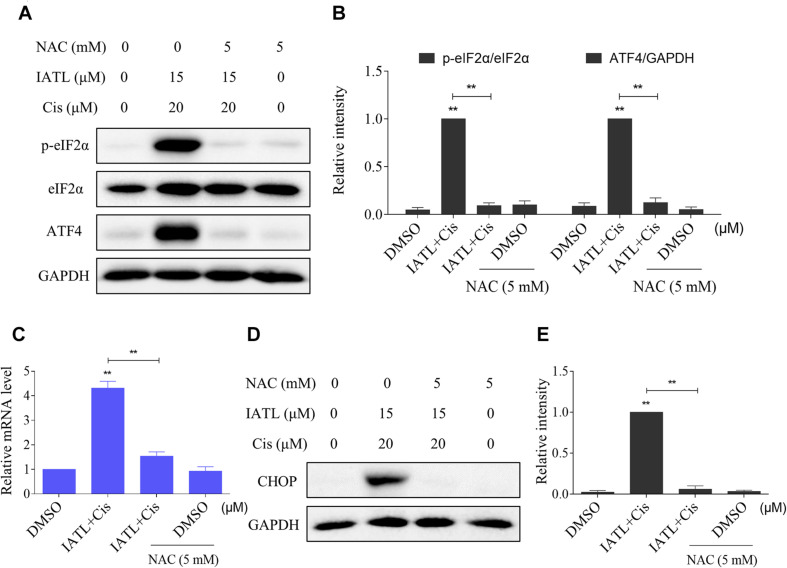
IATL and cisplatin co-operate to activate ROS-dependent ER stress. **(A,B)** DU145 cells were preincubated with NAC (5 mM) for 2 h before exposure to the combination of IATL (15 μM) and cisplatin (20 μM); the expression of p-eIF2α and ATF4 was measured by Western blot. **(C)** DU145 cells were preincubated with NAC (5 mM) for 2 h; the mRNA level of CHOP was determined by qRT-PCR after being treated with IATL (15 μM) and cisplatin (20 μM) combination for 6 h. **(D,E)** DU145 cells were preincubated with NAC (5 mM) for 2 h before exposure to the combination of IATL (15 μM) and cisplatin (20 μM) for 12 h; the expression of CHOP was measured by Western blot. **p < 0.01.

We then determined the relationship of ROS accumulation and ER stress activation in DU145 cells. The Western blot analyses showed that pretreatment with NAC significantly blocked the combined treatment-induced increase in the expression levels of p-eIF2α and ATF4 (Figures 4A,B). Furthermore, the combined treatment-induced CHOP was also reversed by NAC pretreatment (Figures 4C–E). Collectively, these results suggest that IATL and cisplatin combination treatment activated ER stress response in a ROS-dependent manner.

### IATL and Cisplatin Combination Activates JNK Signaling Pathway

**FIGURE 5:**
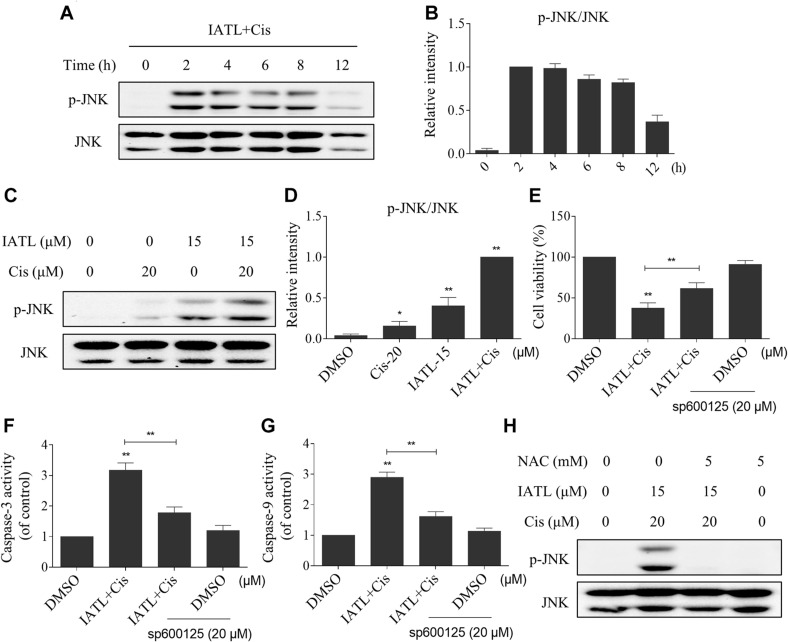
IATL and cisplatin co-operate to induce JNK phosphorylation in prostate cancer cells. **(A,B)** DU145 cells were treated with the combination of IATL (15 μM) and cisplatin (20 μM) for the indicated time periods; the expression of p-JNK and JNK was measured by Western blot. **(C,D)** DU145 cells were treated with IATL (15 μM) or cisplatin (20 μM) alone or their combination (15 μM IATL and 20 μM cisplatin); the expression of p-JNK and JNK was measured by Western blot. **(E)** DU145 cells were preincubated with SP600125 for 2 h; cell viability was detected by an MTT assay after being treated with the combination of IATL (15 μM) and cisplatin (20 μM) for 24 h. **(F,G)** DU145 cells were preincubated with SP600125 for 2 h before being treated with the combination of IATL (15 μM) and cisplatin (20 μM) for 20 h; caspase-3 and caspase-9 activity was measured by an assay kit. **(H)** DU145 cells were preincubated with NAC (5 mM) for 2 h before being treated with the combination of IATL (15 μM) and cisplatin (20 μM); the expression of p-JNK and JNK was measured by Western blot. *p < 0.05, **p < 0.01.

The JNK signaling pathway can be activated in response to various stimuli, including cytokines and oxidative stress (Wang K. et al., 2019; Wang L. et al., 2019). Therefore, we examined whether the JNK signaling pathway was activated in prostate cancer cells after being treated with IATL and cisplatin. The time-course experiment showed that IATL and cisplatin combination significantly increased the expression of p-JNK (Figures 5A,B). Moreover, the addition of IATL in combination with cisplatin resulted in a more significant increase in the expression level of p-JNK as compared with the agent alone (Figures 5C,D). To further confirm the role of the JNK signaling pathway in the combined treatment-induced cell death, cells were pretreated with SP600125 (a specific JNK inhibitor) for 2 h and combined treatment with IATL and cisplatin. The results showed that SP600125 attenuated the combined treatment-induced cell death as well as the activation of caspase-3 and caspase-9 (Figures 5E–G), indicating that the JNK signaling pathway is essential for the synergistic effects of IATL and cisplatin. Notably, the elevated expression of p-JNK was fully abolished by NAC pretreatment (Figure 5H), suggesting that the activation of the JNK pathway is due to ROS accumulation in prostate cancer cells.

### IATL and Cisplatin Synergistically Inhibit Tumor Growth in Nude Mice

**FIGURE 6:**
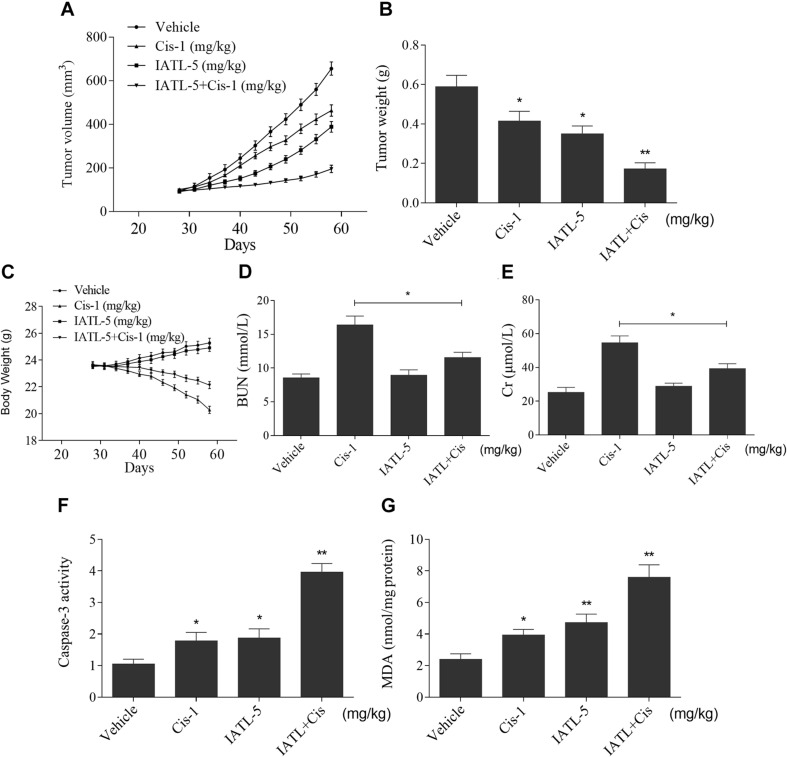
IATL and cisplatin synergistically inhibit tumor growth in nude mice. **(A,B)** IATL in combination with cisplatin markedly suppressed tumor volume **(A)** and tumor weight **(B)**. **(C)** The body weight of nude mice. **(D,E)** The levels of blood urea nitrogen (BUN) and creatinine (Cr). **(F)** Caspase-3 activity in the tumor tissues. **(G)** MDA levels in the tumor tissues. *p < 0.05, **p < 0.01.

To investigate whether IATL can sensitize cancer cells to cisplatin *in vivo*, DU145 cells were inoculated subcutaneously into the right flank region of nude mice. The nude mice were randomly divided into four groups and were treated with vehicle, IATL (5 mg/kg), cisplatin (1 mg/kg), or the combination of IATL (5 mg/kg) and cisplatin (1 mg/kg) by intraperitoneal injection once every 3 days. The results showed that IATL and cisplatin combination significantly inhibited tumor growth and weight as compared with the agent alone (Figures 6A,B). Interestingly, IATL administration protected against cisplatin-induced body weight loss and decreased blood urea nitrogen (BUN) and creatinine (Cr) levels (Figures 6C–E). Consistent with *in vitro* results, the activity of caspase-3 was significantly increased in the combination group (Figure 6F). Importantly, IATL and cisplatin combination markedly increased the MDA level, which is a presumptive measure of ROS-mediated injury, in tumor tissues (Figure 6G). Taken together, our data indicate that IATL sensitized prostate cancer cells to cisplatin *in vivo* by inducing oxidative stress.

## Discussion

Cisplatin is a standard chemotherapeutic agent used to treat prostate cancer, but its clinical use is restricted owing to its limited efficacy and toxic side effects (Hager et al., 2016). Recently, combination therapies have been developed to increase the sensitivity of cancer cells to conventional chemotherapy drugs (Zhang et al., 2019; Chung et al., 2020). Natural products from plants are, in general, safe and have low toxicity, making them ideal chemotherapy sensitizers for the treatment of cancer (Zubair et al., 2020). In this study, we examined whether IATL could synergize with cisplatin in prostate cancer cells. Our results showed that IATL and cisplatin combination significantly suppressed cell growth and induced apoptosis in prostate cancer cells. IATL and cisplatin combination increased the levels of intracellular ROS, which leads to activation of ER stress and the JNK signaling pathway in prostate cancer cells. Importantly, IATL and cisplatin combination significantly reduced the tumor size and weight in a xenograft model. Interestingly, IATL administration also decreased the side effects associated with cisplatin, but the precise mechanism remains unclear. Further experiments are required to fully clarify the underlying mechanisms.

ROS are metabolic byproducts of aerobic respiration and are responsible for regulating various cellular functions. Cancer cells have increased rates of ROS generation due to distorted metabolism and exaggerated replicative. Therefore, cancer cells with increased ROS levels tend to be more vulnerable to agents that promote ROS production (Trachootham et al., 2009; Yang et al., 2016). Numerous studies have shown that natural products can exert their antitumor effects by inducing ROS generation (Hseu et al., 2019; Liu et al., 2019). In addition, some anticancer drugs like sorafenib and osimertinib are known to induce ROS generation as part of its mechanism of action (Tang et al., 2017; Heslop et al., 2020). In our study, we showed that IATL and cisplatin combination significantly increased ROS levels in prostate cancer cells, and pretreatment with the ROS scavenger NAC significantly blocked the increase in ROS production and cell apoptosis, indicating that the elevation of intracellular ROS levels is crucial for the synergistic effects of IATL and cisplatin. Moreover, we found that IATL and cisplatin combination activated the JNK signaling pathway, and pretreatment with the JNK inhibitor SP600125 attenuated the combined treatment-induced cell death, indicating that the JNK signaling pathway is essential for the synergistic effects of IATL and cisplatin. Notably, the elevated expression of p-JNK was significantly reversed by NAC pretreatment, suggesting that activation of the JNK signaling pathway is a downstream event of ROS generation. These data suggest that IATL-induced ROS plays a critical role in cisplatin sensitivity, and provide a new strategy for treating cancer with IATL in combination with existing ROS-inducing drugs or physical treatments.

The ER is involved in protein synthesis, folding, and modification. ER stress occurs when there is accumulation of misfolded proteins in the ER lumen. It has been reported that isobavachalcone, δ-tocotrienol, and CWP232291 could induce ER stress-mediated apoptosis in prostate cancer cells (Li et al., 2018; Fontana et al., 2019; Pak et al., 2019). In accordance with these studies, we found that IATL and cisplatin combination significantly increased the levels of ER stress-related proteins, such as p-eIF2α, ATF4, and CHOP in DU145 and PC-3 cells. Additionally, our results showed that CHOP knockdown attenuated the cytotoxicity activity of IATL and cisplatin. Recent studies showed that ROS have emerged as a crucial regulator of ER stress-induced apoptosis in cancer cells (Zhao et al., 2019; Hu et al., 2020). Our findings showed that pretreatment with NAC significantly blocked the combined treatment-induced increase in the expression levels of p-eIF2α, ATF4, and CHOP, suggesting that ER stress activation induced by the combination of IATL and cisplatin is dependent on intracellular ROS accumulation. These results indicate that activating ROS-mediated ER stress is a useful antitumor strategy.

In conclusion, the present study is the first to demonstrate that IATL and cisplatin induce significant synergistic effects in prostate cancer cells. The synergism of IATL with cisplatin was based on increased accumulation of intracellular ROS. Our findings suggest that the combination of IATL and cisplatin could be a novel therapeutic strategy for treating prostate cancer.

## Data Availability Statement

The original contributions presented in the study are included in the article/supplementary material, further inquiries can be directed to the corresponding author/s.

## Ethics Statement

The animal study was reviewed and approved by the Institutional Animal Care and Use Committee of Wenzhou Medical University.

## Author Contributions

HH and PL carried out most of the experiments. XY, FZ, QL, and KW analyzed the data. WC designed and analyzed experiments, supervised the study, and wrote the manuscript. All authors contributed to the article and approved the submitted version.

## Conflict of Interest

The authors declare that the research was conducted in the absence of any commercial or financial relationships that could be construed as a potential conflict of interest.

## Abbreviations

IATL: isoalantolactone
NAC: N-acetylcysteine
ROS: reactive oxygen species
CI: combination index
ER stress: endoplasmic reticulum stress.
